# Treatment of agrammatism in oral and written production in patients with Broca’s aphasia The use of implicit and explicit learning

**DOI:** 10.1590/1980-57642020dn14-020002

**Published:** 2020

**Authors:** Marcela Lima Silagi, Olavo Panseri Ferreira, Isabel Junqueira de Almeida, Janaina de Souza Simões, Sueli Aparecida Zampieri, Beatriz Raz Franco de Santana, Letícia Lessa Mansur

**Affiliations:** 1 Speech Pathologist. Department of Physical Therapy, Speech, and Occupational Therapy of the School of Medicine - University of Sao Paulo, SP, Brazil.

**Keywords:** aphasia, agrammatism, rehabilitation, explicit learning, implicit learning, afasia, agramatismo, reabilitação, aprendizado explícito, aprendizado implícito

## Abstract

Several approaches to the rehabilitation of agrammatism use implicit and explicit learning methods.

**Objective:**

To verify the effect of adapted Mapping Therapy and ORLA methods (explicit versus implicit learning) on the oral and written production in spontaneous language among agrammatic patients with Broca’s aphasia.

**Methods:**

Six individuals were submitted to Mapping Therapy and ORLA (Oral Reading for Language in Aphasia) treatments. Samples of oral and written production from a picture description task were compared pre and post-treatment.

**Results:**

In Mapping Therapy, the patients presented better performance after the training for the variables related to written production: number of words, nouns, verbs, closed-class words, and number of complete sentences. Regarding oral output, the patients had similar performance before and after the therapeutic process. In ORLA, the patients presented a significant difference before and after the therapeutic process in the variables related to oral production, increasing the number of words, number of verbs, and speech rate. There was no difference in pre and post-treatment performance in written production.

**Conclusion:**

Both implicit and explicit learning can be used in the treatment of agrammatism. Mapping Therapy was more effective for the treatment of agrammatism in written production, while ORLA was more effective for the agrammatism in oral production.

## Resumo.

Várias abordagens terapêuticas para a reabilitação do agramatismo utilizam métodos implícitos e explícitos de aprendizado.

**Objetivo:**

Verificar o efeito dos métodos adaptados Mapping Therapy e ORLA (aprendizagem explícita *versus* implícita) na produção oral e escrita de discurso em pacientes agramáticos com afasia de Broca.

**Métodos:**

Seis indivíduos foram submetidos aos tratamentos Mapping Therapy e ORLA. Amostras das produções oral e escrita na tarefa de descrição da prancha foram comparadas antes e após o tratamento.

**Resultados:**

No método Mapping Therapy, os pacientes apresentaram melhor desempenho após o tratamento nas seguintes variáveis ​​relacionadas à produção escrita: número de palavras, substantivos, verbos, palavras de classe fechada e número de frases completas. Em relação à emissão oral, os pacientes mantiveram desempenho semelhante pré e pós processo terapêutico. No método ORLA, os pacientes apresentaram diferença significativa antes e após o processo terapêutico nas variáveis ​​relacionadas à produção oral, com aumento no número de palavras, número de verbos e velocidade de fala. Não houve diferença no desempenho pré e pós tratamento na produção escrita.

**Conclusão:**

Tanto a aprendizagem implícita como a explícita têm o potencial de serem usadas no tratamento do agramatismo. A Mapping Therapy foi mais eficaz no tratamento do agramatismo na produção escrita, enquanto o ORLA foi mais eficaz para o agramatismo na produção oral.

A grammatism is one of the most frequent manifestations in aphasia. It is conceptualized as a morpho-syntactic deficit, that affects the production and comprehension of language. The main characteristics are short phrase length, simplified syntax, omission and/or substitution of grammatical morphemes, such as plural markers and closed-class words (prepositions, conjunctions, articles, and connectives), and omission of verbs.^1^^,^ 2

Both oral and written language can be affected by agrammatism. However, studies on written production are scarce. Some findings show that spoken and written production typically shows similar error patterns.^3^^,^ 4

The literature reports several therapeutic approaches to the rehabilitation of agrammatism. Two major treatment approaches are described for impaired sentence production: Mapping Therapy^5^ and Treatment of Underlying Forms (TUF).^6^ Both methods are focused on grammatical sentence structure, through training of the linguistic properties of verbs (arguments and thematic roles). While Mapping Therapy addresses a hierarchy of complexity from the simplest to the most complex sentences, TUF does the opposite - it first works with the most complex sentences in an attempt to promote more generalization.^7^

Another method for the treatment of agrammatism described in the literature is the Sentence Production Program for Aphasia (SPPA).^8^ This method aims is to expand the repertoire of grammatical structure of sentences based on a hierarchized training ranging from repetition to spontaneous production in context, with the aid of figures.

A method used in speech therapy - Oral Reading for Language in Aphasia (ORLA)^9^ - can also be used to treat agrammatism. This is a method based on the systematic reading of sentences through a hierarchy of steps with increasing complexity, based on neuropsychological models of reading, by providing practice on phonological and semantic reading routes.

The methods described above, besides differing in terms of working with different grammatical structures, are also distinguished by the learning model required for training: more implicit or more explicit. Implicit learning is defined as learning without intention and conscious awareness, as opposed to explicit learning, that is related to nondeclarative memory systems, when memory underlies non-conscious learning processes such as priming, classical conditioning, and procedural memory for skills and habits.^10^

From this standpoint, Mapping Therapy and TUF could be considered methods that predominantly recruit explicit learning, since the patient is exposed to the syntactic rules in a conscious way, whereas the SPPA and ORLA methods are more associated with implicit learning, since the syntactic rules are not exposed to patients and are internalized through repeated training.

Previous studies have shown positive results with Mapping Therapy and TUF methods for rehabilitation of agrammatism.^5^^,^ 6 On the other hand, implicit learning procedures have the potential to greatly enhance language training because they can lead to less effortful language rehabilitation strategies. However, these learning process procedures have received little attention in the aphasia rehabilitation literature. Implicit and explicit learning in patients with agrammatism was previously explored by Schuchard and Thompson.^11^ however, the authors focused on the learning of word series by auditory input. We found no studies on the use of both explicit and implicit learning specifically for agrammatism therapy.

Considering the scarcity of studies about agrammatism therapy in Brazil (see Silagi, Hirata and Mendonça),^12^ the present study aims to verify the effect of Mapping Therapy and ORLA methods on oral and written production in spontaneous language among agrammatic patients with Broca’s aphasia, from the perspective of explicit versus implicit learning.

## METHODS

### Participants

The sample was composed of six subjects recruited from a Speech-Language Pathology and Audiology ambulatory service. The participants were required to meet the following criteria to participate in the study: age ≥18 years, ≥ 4 years of education, right-hand dominance (as determined by the Edinburgh Inventory),^13^ Brazilian Portuguese as native language, normal or corrected vision and hearing, absence of psychiatric history or neurological disorders other than stroke, and chronic unilateral lesions in the left hemisphere due to an ischemic vascular etiology, with time post onset of >12 months, documented by neuroimaging (computerized tomography or magnetic resonance imaging).

The subjects were submitted to the Boston Diagnostic Aphasia Examination (BDAE - short form)^14^ in its adapted Brazilian Portuguese version^15^ for comprehensive language assessment and to the Northwestern Assessment of Verbs and Sentences (NAVS)^16^ also adapted to Brazilian Portuguese^17^ - verb naming and sentence production subtests for the evaluation of agrammatism.

All subjects received a diagnosis of Broca’s aphasia, with satisfactory performance in oral comprehension, reading aloud and reading comprehension for single words and simple phrases. Oral expression was marked by effortful speech and agrammatic manifestations, such as telegraphic speech and grammatical simplification. Verb naming and sentence production deficits were prevalent for all participants. The oral and written output consisted of isolated words and simple phrases and sentences, with omission of grammatical morphemes, closed-class words and verbs.

The subjects were aged between 46 and 74 years (mean=58/SD=9.4), had formal education of between 5- and 15 years (mean=10.3/SD=4.3), and had unilateral extensive lesions in the middle cerebral artery region in the left hemisphere, with time post onset of >12 months (mean=38.5/SD=12.6). Regarding the severity of aphasia, one patient presented mild aphasia, three patients were moderate, and two had severe aphasia. Aphasia severity was defined using the aphasia severity score scale,^14^ according to the following characteristics: mild aphasia (patient can discuss almost all the problems of everyday life with little or no assistance. The reduction in speech and/or comprehension, however, makes conversation on certain topics impossible); moderate aphasia (conversation about familiar topics is possible with the help of the interlocutor. There are frequent failures to convey the idea, but the patient is able to share responsibility for communication); and severe aphasia (all communication is through fragmented expression; great need for inference, questioning or guesswork on the part of the interlocutor. The information that can be exchanged is limited and the listener takes responsibility for the communication). Four patients had moderate apraxia of speech. Table 1 shows the demographic and clinical data of the sample.

**Table 1:** Demographic and clinical characteristics of the sample.

Patient	Age	Gender	Education (years)	Site of lesion	Time post-onset (months)	Aphasia severity
P1	52	F	15	Frontoparietal and internal capsule	61	moderate
P2	46	M	5	Frontoparietal and insula	31	mild
P3	50	M	7	Frontoparietal and insula	29	moderate
P4	65	M	15	Parietal and internal capsule	26	moderate
P5	61	M	5	Temporoparietal	34	severe
P6	74	M	15	Frontotemporoparietal	50	severe

This study was approved by the Ethics Committee for the Analysis of Research Projects of the institution where it was conducted, under protocol number CAAE179/16. All subjects signed the Free and Informed Consent form to participate in the study.

### Intervention

The participants were submitted to adapted Mapping Therapy and ORLA treatments. The treatment was always in the same order: Mapping Therapy followed by ORLA, with a four-month interval between interventions. The patients did not undergo any structured language therapy in the interval between interventions. In the first two months there was a recess period and in the other two months they participated in unstructured conversation groups. For both methods, one training session was used to familiarize patients with the type of activity, and 15 1-hour weekly therapy sessions were conducted, for a total of 16 sessions. The therapies were applied in groups.

#### Adapted mapping therapy

Seven two-argument verbs (cut, break, kick, tidy, and sew) and four semantically reversible verbs (bite, kiss, hug and lick) were selected. Argument is the designation assigned to the complement selected by the verb or provided for in its argument structure. Arguments of the verb are the syntagmas that perform the function of subject, direct object, indirect object, and complement.^18^ A sentence is semantically reversible when both actors in the sentence could conceivably carry out or be affected by the action of the verb. For example, in the sentence *“The man is hugging the woman”*, both the man and the woman can be the “doer” (i.e., the agent) of the action hugging, or the receiver (i.e., the theme) of the hugging.^19^

**Figure 1:**
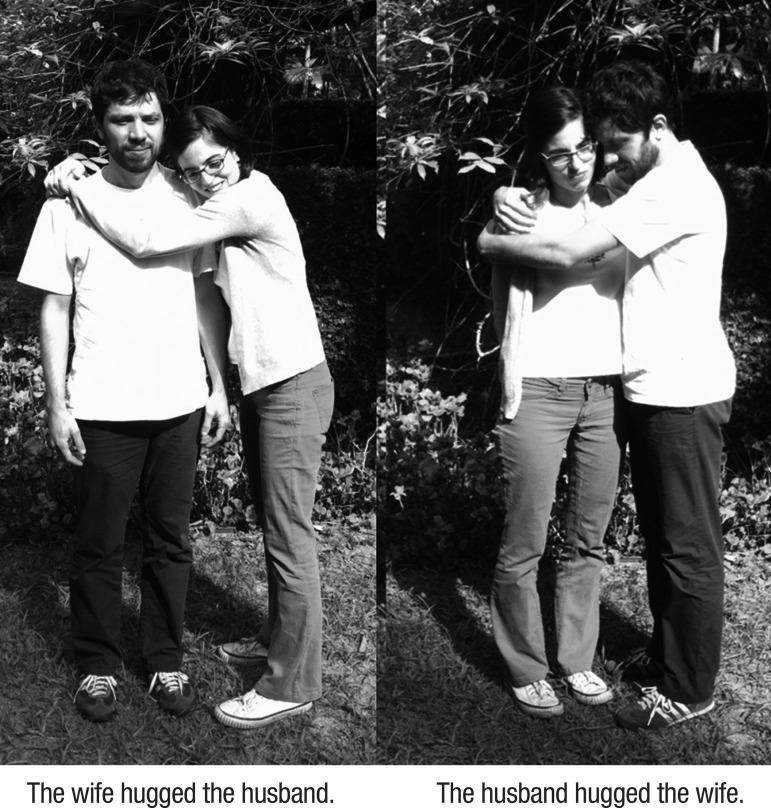
Adapted Mapping Therapy.

The sentences were worked with according to these steps: (1) Each patient received colorful cards representing different classes and pictures corresponding to the actions (only one figure was provided at a time); (2) The patients were instructed to produce sentences aloud, according to the model of the syntactic structures from the written stimulus; (3) The patients had to copy the written model and highlight the different elements of sentences with corresponding colored pens (different colors for verbs, subjects and objects); (4) The cards were removed and the patients had to try and say the sentence again, in the absence of the model; (5) The patients were stimulated to construct new sentences aloud retaining the two arguments but changing the verb; (6) The patients had to produce one sentence or more aloud changing the arguments, with the help of the therapist; (7) The patients had to say the sentence to the group. An example is given in Figure 1.

#### Adapted ORLA

**Figure 2:**
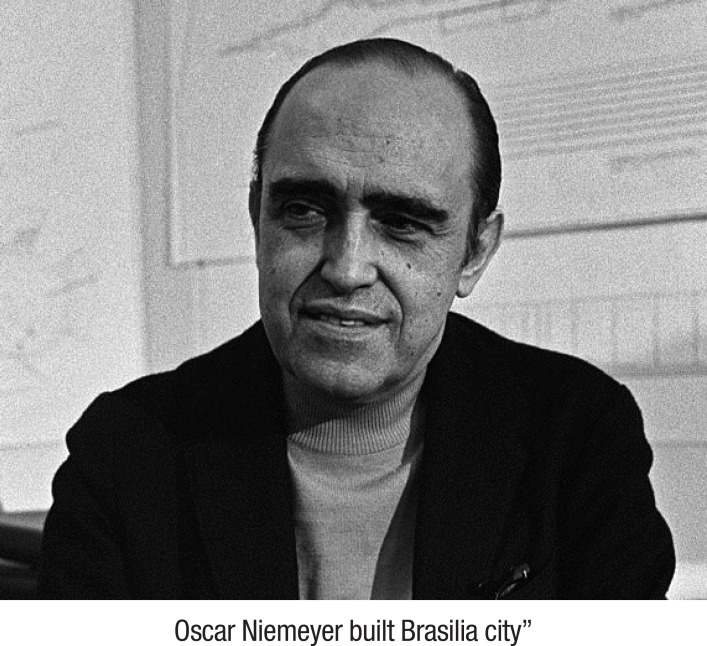
Adapted ORLA.

Simple 3- to 5-word sentences at a ﬁrst-grade reading level of ORLA, related to the biographies of famous people, were produced in a systematic training, following the steps: (1) The therapist read aloud to the patients, pointing to each word as he or she read along; (2) The therapist read aloud to the patients again, pointing to each word, and the patients also had to point to each word, but silently; (3) The therapist and the patients read the sentence aloud together and pointed to each word (the clinician adjusts the rate and volume of the oral reading according to the specific patient (e.g., reading a little ahead of the patient so he or she is able to hear the initial phonemes of the words; decreasing volume as the patient requires fewer cues); (4) For each sentence, the therapist pointed to a word for the patients to read aloud alone. Words were content words (e.g., nouns, verbs) or function words (e.g., pronouns, prepositions, conjunctions); and; (5) The patients read the whole sentence aloud again in unison with the therapist. An example is given in Figure 2.

### Data analysis

In order to verify the effect of the training on each method, samples of oral and written output from the Boston Cookie Theft picture description, a task from the Boston Diagnostic Aphasia Examination, were compared before and after treatment. The following variables were used: number of words, number of nouns, number of verbs, number of closed-class words, number of complete sentences and sentence length. In addition, the speech rate (words per minute) was measured in the oral production task.

Two baseline probe scores were conducted (pre and post-treatment). For the descriptive analysis, the mean and standard deviation were calculated for each variable on oral and written production from the Boston Cookie theft picture description task. The Mann-Whitney test was used to compare pre- to post-treatment group mean scores. A statistical significance level of 0.05 was adopted.

## RESULTS

Table 2 shows the performance of the participants for the Mapping Therapy method.

**Table 2:** Performance of subjects at pre and post-treatment using Mapping Therapy.

Variables	Oral production				Written production		
	Pre	Post	p		Pre	Post	p
Number of words	28.5 (27.9)	24.8 (19.2)	ns		9.0 (7.6)	13.2 (11.8)	0.015
Number of nouns	8.5 (7.0)	7.3(4.3)	ns		3.1(3.2)	4.8(4.5)	0.047
Number of verbs	5.3 (4.4)	4.8 (3.6)	ns		1.8(2.1)	3.2(3.7)	0.007
Number of closed-class words	9.1 (11.5)	9.3(10.7)	ns		2.7 (2.9)	4.1(4.4)	0.037
Number of complete sentences	2.6 (2.6)	2.0(2.0)	ns		1.2(1.3)	2.0 (2.0)	0.047
Sentence length	3.0 (2.5)	4.1 (4.7)	ns		2.5 (2.5)	3.8 (4.0)	ns
Speech rate (words/minute)	15.6 (12.8)	14.3 (8.3)	ns		-----	-----	-----

Mann-Whitney; ns: not significant.

On the Mapping Therapy method, the patients had better performance after the training for the following variables related to written production: number of words, number of nouns, number of verbs, number of closed-class words, and number of complete sentences. Regarding oral production, the patients had similar performance before and after the therapeutic process.

Table 3 shows the performance of the participants using the ORLA method.

**Table 3:** Performance of subjects at pre and post-treatment using ORLA.

Variables	Oral production				Written production		
	Pre	Post	p		Pre	Post	p
Number of words	13.8 (10.3)	22.0 (13.5)	0.018		4.1 (3.6)	4.5 (3.7)	ns
Number of nouns	9.3 (5.0)	11.0(4.9)	ns		2.3 (1.2)	2.9 (2.2)	ns
Number of verbs	1.9 (3.9)	3.7 (3.9)	0.017		0.7 (1.2)	0.9 (1.3)	ns
Number of closed-class words	4.6 (13.6)	3.1(3.9)	ns		1.8 (1.7)	1.8 (1.7)	ns
Number of complete sentences	0.8 (1.8)	0.6(1.4)	ns		0.8 (1.4)	0.9 (1.3)	ns
Sentence length	1.5 (2.0)	2.5 (3.5)	ns		1.6 (1.7)	1.8 (1.8)	ns
Speech rate (words/minute)	12.1 (9.8)	18.7 (13.6)	0.048		-----	-----	-----

Mann-Whitney; ns: not significant.

On the ORLA method, the patients showed a statistically significant difference in performance before and after the therapeutic process for the variables related to oral production, with an increase in the number of words, number of verbs, and speech rate. There was no difference in pre and post-treatment performance in written production.

## DISCUSSION

Considering the interaction of explicit and implicit learning in agrammatism, the aim of this study was to verify the effect of Mapping Therapy and ORLA methods on the oral and written production in spontaneous language of six agrammatic patients with Broca’s aphasia.

First, it is important to note that participants were in the chronic stage of aphasia, when spontaneous language improvement is likely to be very small,^20^ and therefore the therapeutic effects *per se* are more evident Another point is the variability in lesion location found among patients. Importantly, our inclusion criteria were based on the participants’ linguistic profile rather than on lesion properties. Lesion analyses showed damage in frontal and posterior perisylvian regions, also extending to subcortical regions, a feature that has been found in other patients with Broca’s aphasia, including those studied by Broca himself (see Dronkers et al.^21^). Regarding the severity of aphasia, our sample consisted predominantly of more severe cases, and also presented comorbidities such as apraxia of speech.

Our results showed improvement of written production (number of words, number of nouns, number of verbs, number of closed-class words, and number of complete sentences) after training with Mapping Therapy. By contrast, the patients improved agrammatism in oral language (number of words, number of verbs, and speech rate) after training with ORLA.

A key point for discussion are the reasons why Mapping Therapy (despite being based predominantly on oral production) improved agrammatism in the written task, and why ORLA (despite being based predominantly on reading) improved agrammatism in speech.

A possible explanation is the type of learning required in each method. Although Mapping Therapy requires some implicit learning for the generalization of syntactic rules, the use of colored cards and highlighted words to link thematic role to subject and object positions activate the conscious analysis of the statements, favoring predominantly explicit learning. In this process there is greater recruitment of directed attention to specific grammatical aspects, favoring the improvement of morpho-syntactic abilities (number of verbs, number of closed-class words, and number of complete sentences).

On the other hand, ORLA improves sentence production through systematic reading training, without the need to make the syntactic rules explicit, which favors more implicit learning. This occurs through the frequency of the same stimulus repetition. Although the ORLA method did not lead to improvement of closed-class words or syntactic structure, there was an improvement in the use of verbs, an essential prerequisite for sentence construction. In parallel, the patients achieved greater speech fluency (increased number of words and speech rate). In addition, reading in unison with the therapist promotes real-time feedback on output, while allowing adjustments to the speech rate, which may be beneficial for speech apraxia. These results were predicted by the authors of the ORLA.^9^

Other reasons for this dissociation can be explained by the different sample profiles in previous studies. Schwartz et al.^5^ used Mapping Therapy in eight chronic non-fluent aphasics and found improvement on predicted measures of sentence production. However, the best outcomes were seen in patients with relatively pure agrammatism. Patients with more severe and complicated impairments had poorer outcomes. Studies using the ORLA also showed different patterns of improvement. Cherney^9^ examined the efficacy of ORLA for 25 individuals with chronic nonfluent aphasia of varying severity levels. Western Aphasia Battery (WAB)^22^ measures were used to calculate therapeutic efficacy. For patients with severe aphasia, medium effect sizes were obtained on reading subtests only. The moderate aphasia group improved on discourse measures only, and for the mild-to-moderate aphasia group, both the discourse and writing subtests improved.

Given that moderate and severe cases predominated in the present sample, our results agree with the studies cited above, in which there was no significant improvement in oral agrammatism with Mapping Therapy in patients with more severe impairments and presence of other comorbidities, but positive results were attained in oral emission using the ORLA for this same severity profile.

Another point to be discussed is the overlapping and generalization effect across the different language modalities during aphasia recovery, due to cross model transfer of competence. Some studies show effects of reading-based therapies on generalization for oral production and vice-a-versa. Katz and Wertz^23^ examined the effects of computer-provided reading activities on language performance in chronic aphasic patients. The patients submitted to the computer reading treatment displayed significant improvement on the Porch Index of Communicative Ability “Overall” and “Verbal” modality percentiles and on the Western Aphasia Battery Aphasia “Quotient” and “Repetition” subtest. Similarly, Singh and Pauranik^24^ found that the simultaneous use of reading and writing approaches was useful in improving verbal skills. One patient was submitted to both reading and writing tasks from word to simple sentence level. Generalization to oral output was tested in picture confrontation naming for nouns, verbs and spontaneous speech during picture description, and a narrative task. The results showed that performance for functional communication aphasic quotient and mean length of utterances improved significantly, along with reading and writing skills. Similarly, Orjada and Beeson^25^ found that concurrent treatment for reading and spelling in aphasia was able to improve oral language performance, with increase in grammatical complexity of spoken language.

The study limitations include the small number of patients and the lack of detail on the agrammatic profile of each patient. Regarding perspectives, the creation of a hybrid therapeutic program is envisaged that explores the method advantages to stimulate both implicit and explicit learning for patients with different degrees of agrammatism severity.

In conclusion, this study showed that the multiple components of agrammatism (oral and written) can be explored in order to achieve better results in the therapeutic process. The results obtained with the use of Mapping Therapy were more effective for the treatment of agrammatism in written production, while ORLA was more effective for agrammatism in oral production. Therefore, both implicit and explicit learning have the potential to be used in the treatment of language in agrammatic individuals with Broca’s aphasia. These findings highlight the need to investigate methods that integrate different types of learning.
